# Interaction of Different Charged Polymers with Potassium Ions and Their Effect on the Yield Stress of Highly Concentrated Glass Bead Suspensions

**DOI:** 10.3390/ma13071490

**Published:** 2020-03-25

**Authors:** Zichen Lu, Simon Becker, Sarah Leinitz, Wolfram Schmidt, Regine von Klitzing, Dietmar Stephan

**Affiliations:** 1 Department of Civil Engineering, Technische Universität Berlin, 13355 Berlin, Germany; 2 Department of Physics, Technische Universität, 64289 Darmstadt, Germany; 3 Bundesanstalt für Materialforschung und-prüfung (BAM), 12205 Berlin, Germany

**Keywords:** yield stress, free polymer, charge density, depletion force, potassium ions

## Abstract

The interaction of different charged polymers, namely anionic polycarboxylate superplasticizer (PCE) and neutral polyethylene glycol (PEG) with potassium ions, and their effect on the yield stress of highly concentrated glass bead suspension (GBS), were studied under different concentrations of potassium ions ([K^+^]). It was found that, compared to the neutral PEG, the negatively charged PCE can be adsorbed on glass beads (GB), and then decreases the yield stress of GBS. The increasing concentration of free polymer in the interstitial liquid phase with the increased polymer dosage leads to the higher yield stress of GBS, which may be caused by the higher depletion force. In addition, this effect is also related to the charge density of the polymer and the [K^+^] in the solution. Along with the increase in [K^+^], the yield stress of GBS increases significantly with the addition of PCE, but this cannot be observed with PEG, which indicates that potassium ions can interact with negatively charged PCE instead of the neutral PEG. At last, the interparticle forces between two single GB with adsorbed PCE in solutions containing [K^+^] and PCE were measured by colloidal probe atomic force microscopy to better understand the interaction of the charged polymer with counterions.

## 1. Introduction

The rheological performance of cementitious materials plays a significant role in their application at construction sites, which can be adjusted through the addition of different chemical admixtures [1]. Polycarboxylate superplasticizer (PCE), the newest generation of superplasticizer (SP), is nowadays commonly used in concrete, due to its strong dispersing ability at low dosages, which makes it possible to produce concrete with a low water-to-cement ratio (w/c), but with excellent workability. However, for concrete with a low w/c ratio, such as self-compacting concrete (SCC) or ultra-high performance concrete (UHPC), a high dosage of PCE is needed to achieve the desired flowability. In some cases, a large amount of PCE can result in a high concentration of non-adsorbed PCE which are left in the interstitial liquid phase [2,3]. Many studies have been published on the effect of adsorbed PCE on the initial rheological performance of cement paste, which has been well explained by the combination of DLVO theory (named after B. **D**erjaguin, L. **L**andau, E. **V**erwey and T. **O**verbeek) and steric hindrance [4,5]. However, more and more researchers have gradually found that, in addition to the effect of the adsorbed polymers, the free polymers left in the liquid phase also affect the initial rheological performance of cementitious materials. Notably, Liu et al. found that an increased viscosity of cement paste can be observed with an increasing concentration of PCE in the interstitial liquid phase, at a w/c ratio of 0.16 [6]. Additionally, Matsuzawa et al. [7,8] pointed out that the effects of over-dosed PCE on the viscosity of cement paste are closely related to the molecule structure of the PCE. Cement paste with the addition of PCE with lower charge densities shows a lower apparent viscosity even though the total adsorbed amount is lower, while the PCE with a high charge density shows the opposite effect. However, the corresponding effect on the yield stress of cement paste is not mentioned in these studies, which primarily concerns the flowability of cement paste. In addition to negatively charged PCE, the effect of the neutral polymer was also discussed. Lange et al. [9] reported that the flowability of cement paste can be improved by adding non-adsorbing polyethylene glycol (PEG, a component of the PCE side chain). They ascribed this phenomenon to the lubrication effect caused by the non-adsorbing PEG between the adjacent cement grains. A similar effect was found by Jiang et al. [10], but they attributed the mechanism to the shielding effect of PEG on the carboxylate groups contained in PCE molecules, which prevents the agglomeration of cement grains due to the complexation of PCE with calcium ions existing in the pore solution or on the surface of cement grains. However, Bessaies-Bey et al. [11] found a significant increase in the yield stress of cement paste through the addition of PEG, which is described by the occurring depletion force, caused by the increasing polymer concentration in the interstitial liquid phase. 

As illustrated above, on the one hand, no consensus has been reached on how free polymer in the interstitial liquid phase affects the rheological performance of cementitious materials. On the other hand, due to the possible interaction between polymers and ions in solution (the complexation, electrostatic interaction, etc.), the molecular structure of the free polymer should play quite an important role in their effect on the rheological performance of cement paste. Unfortunately, the importance of the molecular structure of the polymer has not yet been well considered to understand its corresponding effect. 

The charge density is an essential factor of molecular structure to influence the performance of SP [12]. From one aspect, a higher charge density of polymer means a stronger adsorption capability of SP on the surface of cement grains, which is beneficial to increase the dispersing capability of SP [13]. Additionally, a higher charge density of free polymer means a stronger interaction with the ions in the interstitial liquid phase, which can form some nanoparticles or clusters in the pore solution of cement paste [14,15]. In summary, the charge density can, to some extent, be seen as the decisive factor in the performance of SP, since any change in the characteristics of the molecular structure, such as side-chain density, side-chain length or the main chain length of PCE, only takes effect after the PCE molecules have been adsorbed [12]. Hence, in this paper, the effect of different charged polymers on the rheological performance of GBS was focused on. 

Yield stress is an important parameter to evaluate the rheological performance of cementitious materials. Lower yield stress usually means a higher spreading capability of concrete. It is commonly observed that the addition of superplasticizer can significantly reduce the yield stress of concrete [16], and the decrease of yield stress is proportional to the adsorbed amount of SP on the surface of cement grains [17]. However, few publications discuss the effect of free polymers with different charge densities on the yield stress of cement paste [10]. At the same time, except for the presence of free polymer, various ions with different concentrations also exist in the interstitial liquid phase of cement paste, such as potassium ions, calcium ions, sulfate ions, etc. Hence, it is necessary to figure out how ions in the interstitial liquid phase interact with free polymers with different charge densities and then affect the yield stress of cementitious materials, which is the aim of our study. 

It should be mentioned here that, due to the complexity of cement grains, which have various mineralogical phases with different charge properties and continuous hydration after contact with water, inert glass beads with a spherical shape and homogeneous surface chemistry were used in this study. In our previous research, the effects of different cations on the yield stress of concentrated GBS in the presence of PCE were studied [18], and in this paper, we mainly focus on the interaction of different charged polymers with cations and their corresponding effect on the yield stress of GBS. Hence, two types of different charged polymers, namely negatively charged PCE and neutral PEG, were selected. Due to the high [K^+^] in the cement pore solution, potassium ions were used as the cations, and the [K^+^] was kept at 100 and 400 mmol/L by adding potassium chloride. At last, in order to better understand the rheological performance, colloidal probe atomic force microscopy (CP-AFM) was applied to directly measure the interaction forces between glass beads with adsorbed PCE in bulk solution, with different amounts of polymer and [K^+^]. 

## 2. Materials and Methods 

### 2.1. Materials

**Figure 1:**
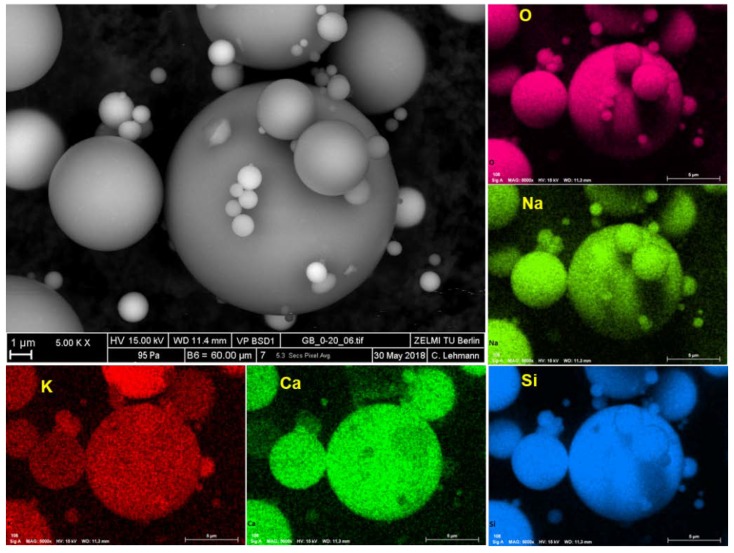
Scanning electron microscope (SEM) pictures of the used glass beads (GB) and the corresponding element analysis.

The glass beads (GB) used in this study were provided by Sigmund Lindner GmbH (Germany). The GB have a spherical shape, as shown in Figure 1. The specific surface area and density of the GB are 0.552 m^2^/g and 2.5 kg/dm^3,^ respectively. The particle size distribution of the GB, their chemical composition, and the ion concentration of the supernatant of the glass bead suspension (GBS), can be found in [18]. It should be mentioned here that, according to Figure 1, in addition to silicon and oxide, many other elements, including calcium, potassium, sodium, are also the components of the GB, which leads to the high concentration of sodium ions (~250 mmol/L) in the supernatant of GBS. Hence, for all the samples prepared for the rheological experiment, a high sodium concentration in the interstitial liquid phase is unavoidable. However, the good news is that nearly the same sodium concentration is obtained in the interstitial liquid phase to the different samples during the rheological experiment. Hence, the existence of sodium ions should not affect our evaluation on the effect of the gradual increase of [K^+^] on the rheological performance of GBS. 

PCE with a Mw of 28,000 Da and a polydispersity index of 2.4 was provided by BASF. The theoretical charge density is 1530 μeq/g. The EO unit number in the side chain of PCE is 18. Two polyethylene glycols (PEG, Merck KGaA Millipore Corporation, Germany) with different molecular weights were used. The molecular weights of the two PEGs are 30,000 Da and 3000 Da, respectively, and they are abbreviated as P30K and P3K. Potassium chloride (KCl), with a pure analytical grade, was used directly without any further purification in the rheological experiment. For the AFM measurements, potassium chloride (≥ 99.5%, p.a.) was purchased from Carl Roth. DI water was used in all experiments in this study. 

### 2.2. Methods

#### 2.2.1. Sample Preparation

A low water-to-GB mass ratio of 0.24 was used for the GBS preparation to prevent segregation and bleeding. Besides, in order to ensure the existence of free polymer in the interstitial liquid phase, the polymer dosage was increased from 0.02 % to 0.8 % by weight of glass beads (bwog). At the fixed polymer dosage of 0.8 %, both the effect of individual polymers (PCE, PEG) and of their mixtures (0.1 % PCE + 0.7 % PEG) on the yield stress of GBS was evaluated, which makes it possible to compare the effect on the yield stress of GBS caused by polymers with different charge densities. The specified process of sample preparation was well described in [18]. The pH of the prepared GBS can be found in Figure S1. 

#### 2.2.2. Rheological Investigation 

The rheological measurements were conducted with an ambient temperature of 20 °C. The rheometer HAAKE RheoStress 600 (Thermo Electron Corporation, Waltham, MA, USA), with a vane-in-cup geometry (Vane rotor FL 29.5 CMC, R = 29.5 mm, order no. 222-1797), was used in the present study. The suspension was measured under rotation with a gradually increasing shear rate of 0 s^−1^ to 8.378 s^−1^. The shear rate was increased every 15 s. After reaching 8.378 s^−1^, the shear rate was gradually decreased in 14 steps. The profile is shown in Figure S2 in the supporting materials. The total measurement time is 7 min. The last five points at each shear rate during the downward steps were recorded and averaged to represent the rheological performance of the GBS. Finally, the change of shear stress τ with the shear rate $\dot{\gamma }$ was probed, and then the yield stress $\tau_{0}$ can be obtained through the fitting using the Herschel Bulkley’s Equation (1). Fits are shown in Figure S3. For each measurement, the same quantity of GBS (600 g) was used.

(1) $$\tau =\tau_{0}+k{\dot{\gamma }}^{n}$$

in which k is the consistency index, and n is the flow index.

#### 2.2.3. Adsorption Measurement 

The adsorption measurement of PCE on the GB surface was well described in [18]. The adsorption of PEG was indirectly measured by the non-adsorbing amount left in solution by a total organic carbon (TOC, TNM-L, Shimadzu, Kyoto, Japan) analyzer. The same sample preparation method, as described in Section 2.2.1, was used, and the supernatant was obtained by centrifugation and filtration (0.45 μm cellulose nitrate syringe filter). The organic carbon concentration in the supernatant was measured and the concentration of PEG in solution was subsequently obtained. Finally, the measured value and the theoretical value without adsorption were compared. 

#### 2.2.4. Zeta Potential 

The zeta potential measurement of GBS with the addition of different polymers was well described in [18]. The electroacoustic method (DT-310, Dispersion Technology, Bedford Hills, NY, USA) was used to measure samples, prepared according to the method illustrated in Section 2.2.1. Each measurement was repeated three times, and the average value was calculated. 

#### 2.2.5. Atomic Force Microscopy

The force measurements were conducted by colloidal probe atomic force microscopy (CP-AFM), in sphere-sphere geometry. The used particles were from the same batch as the GB used for rheological measurements, but diameters between 4 and 6 µm were chosen. The particles at the cantilever (HQ:CSC37:tipless:NoAl, MikroMasch) and at the bottom plate were immobilized using epoxy resin (UHU Endfest 300). The force constants of the cantilevers (391–485 pN/nm) were determined using the Sader method [19]. The solutions for AFM were prepared to fit the bulk solution of samples for the rheological measurements. Hence, only PCE and/ or potassium chloride are in the solution. No other ions dissolved from GB, such as sodium ions or calcium ions, are existing. The PCE concentrations at the dosage of 0.1 % bwog and 0.8 % bwog were 4.08 g/L and 29.04 g/L, respectively. For better comparison, these concentrations will also be denoted as 0.1 % and 0.8 % in the AFM measurement. 

For centering the particles over one another, an inverted optical microscope (Olympus IX71, Olympus Europa SE & CO. KG, Hamburg, Germany) was used, together with an AFM (MFP3D Bio, Asylum Research, Santa Barbara, CA, USA). The recorded raw signals deflection at the photodiode and displacement of the z-piezo were transformed, as described in [20] using Igor (WaveMetrics). Each force curve shows a combination of at least 40 individual force curves, which were averaged by binomial smoothing function (10³ data points). The resulting curves were thinned afterward to show every 1000th data point. The smoothed approach and retraction force curves are consequently shown in filled and open symbols, respectively. For a general comparison of the forces independent of the glass beads curvature, the forces between the surfaces are normalized to the effective radius. The force between two curved surfaces at a separation *d* (*F(d)*) can be transformed into the interaction free energy per unit area W(d) between the corresponding planar surfaces, according to the Equations shown in [21].

(2) $$\frac{F\left(d\right)}{R_{eff}\;}=2\pi W\left(d\right)$$

in which the effective radius can be calculated based on the equation below.

(3) $$R_{eff}=\frac{R_{1}\times R_{2}}{R_{1}+R_{2}}$$

where $R_{1}$ and $R_{2}\;$ are the radii of curvature of the corresponding surfaces. This has been experimentally investigated by [22]. 

It should be noted that, in contrast to the rheological measurements, it is difficult to use fresh systems for each concentration in AFM. Therefore, the systems were prepared in a different method. The system was rinsed first several times by PCE 0.8 % and 400 mM KCl to reach the adsorption of the PCE on the surface of the GB. Afterwards, the system was rinsed by water to get rid of the loosely attached residual PCE. Furthermore, the system was rinsed several times with the corresponding solution of PCE and KCl to reach the corresponding bulk density of the solution before each measurement.

## 3. Results

### 3.1. Effect of Negatively Charged PCE on the Yield Stress

**Figure 2:**
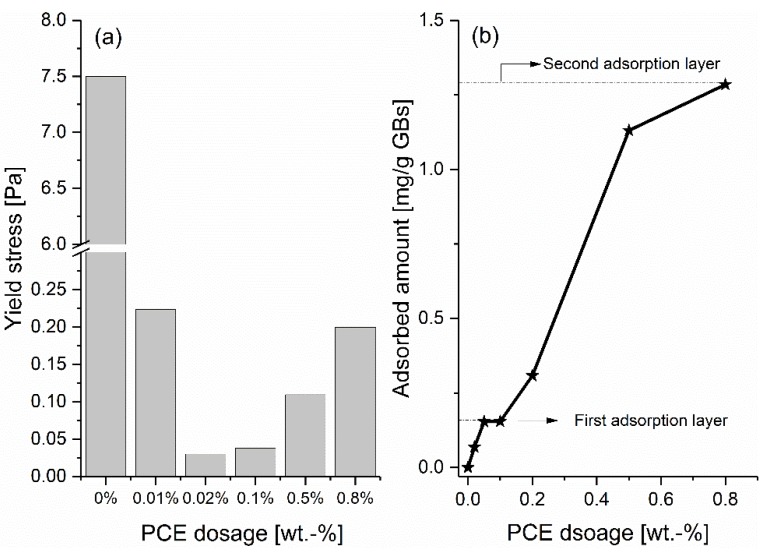
Yield stress of GBS (**a**) [18] and the adsorbed amount of PCE (**b**) along with the increasing PCE dosages.

In order to thoroughly compare the effect of different charged polymers on the yield stress of GBS, it is necessary to first look at how the negatively charged PCE alone affects the yield stress of GBS, with increasing dosages. As shown in Figure 2a [18], compared to the sample without the addition of PCE, the yield stress of GBS firstly decreases and then increases along with the increased dosages of PCE. The minimum yield stress is obtained at PCE dosages between 0.01% and 0.1 %. In order to better understand this phenomenon, the adsorption isotherm of PCE on the surface of GB was measured, and the results are shown in Figure 2b. The plateau at around 0.1 % gives a strong hint for a two-step adsorption process, where a first layer is saturated at this concentration. This plateau occurs in the same PCE concentration regime where the yield stress shows its minimum. Further, PCE adsorption does not affect the dispersion of GBS, which corresponds well with the results of other researchers [23]. Meanwhile, it should be mentioned here that more and more PCE are left in the interstitial liquid phase as free polymer with the increasing PCE dosage. At the dosage of 0.8 %, the PCE concentration in the interstitial liquid phase can be about 28.1 g/L. 

Due to the electrostatic attraction between the negatively charged PCE and the cations in a respective real pore solution, the effect of potassium ions on the yield stress of GBS was investigated. Two typical PCE dosages were selected in this study, namely 0.1 %, at which GBS has low yield stress and the highest measured dosage of 0.8 %, where the yield stress is quite high. 

**Figure 3:**
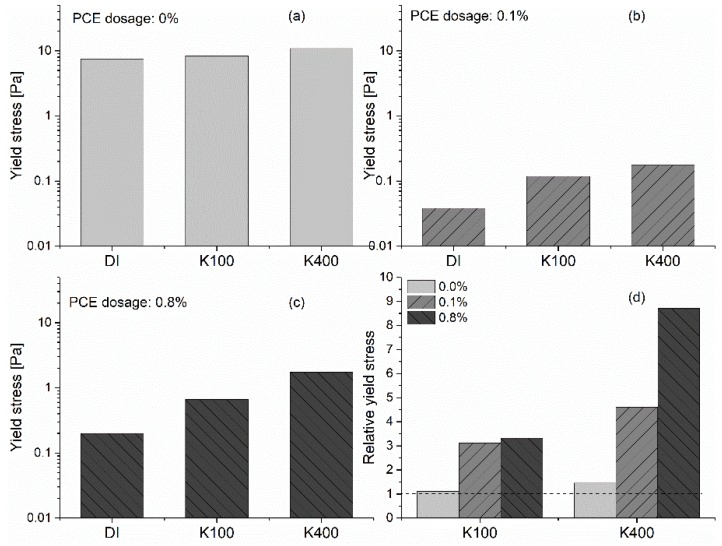
Effect of [K^+^] on the yield stress of GBS with different dosages of PCE; (**a**) 0 %; (**b**) 0.1 %; (**c**) 0.8 %; (**d**) relative yield stress, along with increasing [K^+^].

The effect of [K^+^] on the yield stress of GBS with the addition of PCE at varied dosages is shown in Figure 3. The relative yield stress in Figure 3d is calculated according to the equation shown in Equation (4).

(4) Relative yield stress= τ[K]τDI

in which $\tau_{\left[K\right]}$ is the yield stress of samples at a certain PCE dosage mixed with 100 or 400 mmol/L potassium chloride solution; $\tau_{DI}$ is the yield stress of samples with DI water and different dosages of polymer (PCE or PEG). 

For the reference system without PCE, the increasing [K^+^] has almost no effect on the yield stress of GBS. However, for the samples with the addition of PCE, as shown in Figure 3b,c, a significant increase in yield stress caused by the increasing [K^+^] can be found with the increasing PCE dosage. If we focus on the relative yield stress caused by the increasing PCE dosages with certain [K^+^], a higher relative yield stress can clearly be found for the samples with the increasing PCE dosages. In addition, a higher [K^+^] leads to a higher relative yield stress. The results above indicate a synergistic effect between the negatively charged PCE and potassium ions. The interaction between potassium ions and PCE increases the yield stress of GBS. 

### 3.2. Effect of Neutral PEG on the Yield Stress

**Figure 4:**
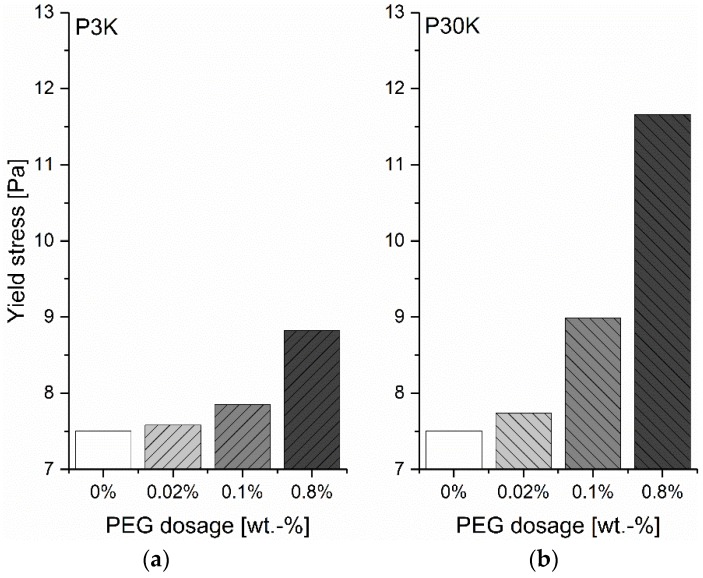
Yield stress of GBS along with the increasing dosages of PEG; (**a**) P3K; (**b**) P30K.

The synergistic effects of cations and negatively charged PCE on the yield stress raise the question of whether the same phenomenon can be observed in the case of neutral polymers. The effect of neutral PEG with different molecular weights on the yield stress of GBS is evaluated in this section. For the samples mixed with DI water, as shown in Figure 4, it is found that the addition of PEG leads to a monotonous increase in the yield stress of GBS for both PEGs with the increasing dosages, which is in contrast to the phenomenon found with the addition of PCE. In addition, the yield stress is much higher for PEG than for PCE of the same molecular weight. At 0.8 %, the yield stress is about 13.5 Pa for P30K, but only 0.2 Pa for PCE. The PEG with a higher molecular weight leads to a higher yield stress. 

**Figure 5:**
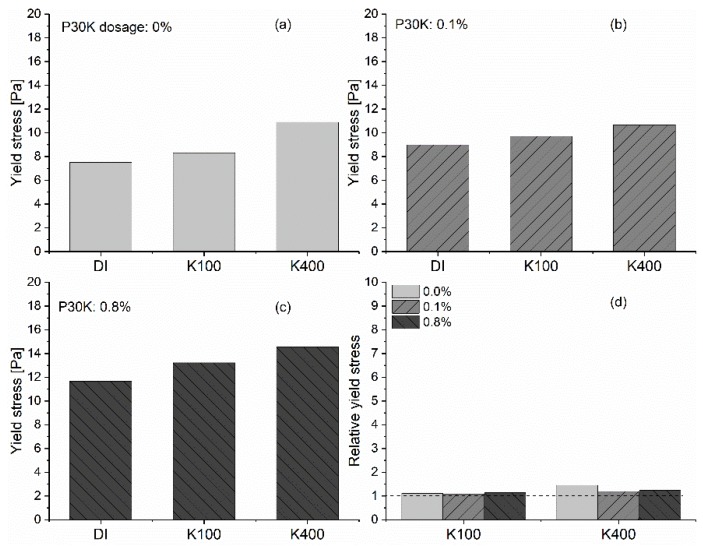
Effect of [K^+^] on the yield stress of GBS with different dosages of P30K; (**a**) 0 %; (**b**) 0.1 %; (**c**) 0.8 %; (**d**) relative yield stress along with increasing [K^+^].

The effect of [K^+^] on the yield stress of GBS with the addition of PEG at varied dosages and the relative yield stress with certain [K^+^] are shown in Figure 5. Contrary to the phenomena found with PCE, the increasing [K^+^] has a limited effect on the yield stress of GBS when PEG is added. The relative yield stress is always around 1 with increasing [K^+^], regardless of the PEG dosage, which indicates that there is almost no interaction between the potassium ions and neutral PEG molecules. 

**Figure 6:**
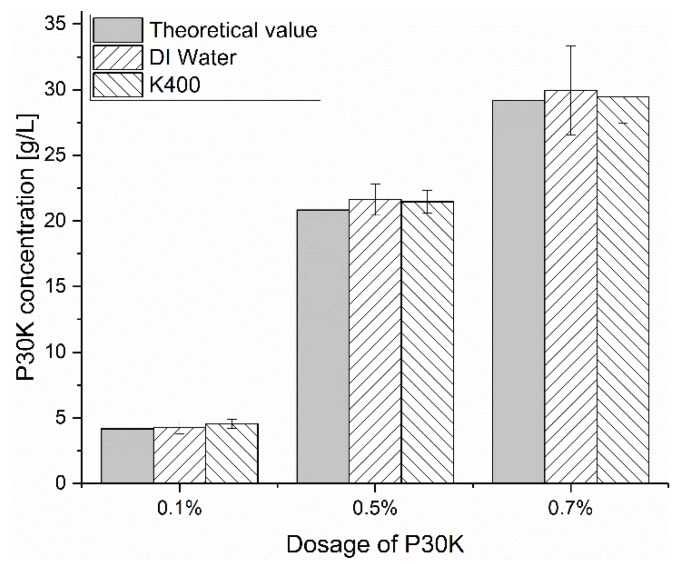
Comparison of the P30K concentration measured in the supernatant of GBS and the calculated theoretical value when no adsorption occurs.

**Figure 7:**
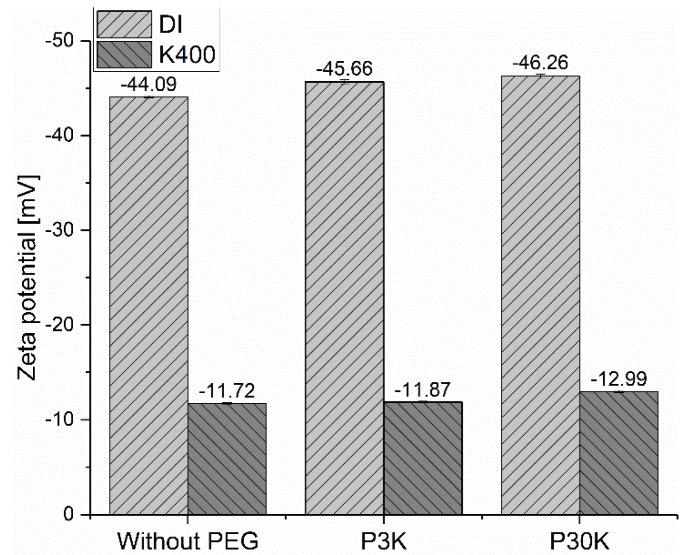
Zeta potential of GBS with the addition of different PEGs.

In some literatures [24,25], it is reported that the addition of PEG is beneficial for the fluidity of nano-sized silica bead suspension, due to the adsorption of PEG on the surface of silica beads [24,25,26,27], which are composed of silicon dioxide. However, this is not the case in our study, even though the main component of GB is still silicon dioxide. In order to find out the reason, the PEG concentration in the interstitial liquid phase was measured by TOC and then compared to the calculated theoretical value, with the assumption of no adsorption. The results are shown in Figure 6. It is found that the measured PEG concentration is nearly the same as the calculated theoretical value, which means that almost no PEG molecules are adsorbed on the surface of GB. In order to verify this result, the zeta potential of GBS with the two types of PEGs at the dosage of 0.8 % was measured. When a non-ionic polymer is added to the dispersion of charge-stabilized particles, the electrical double layer around the particles and the zeta potentials should be altered if the polymer molecules adsorb onto the surface of particles. Normally, the absolute zeta potential value should decrease due to the shift of the slipping plane [28]. However, as shown in Figure 7, the addition of PEG has almost no effect on the measured zeta potential, irrespective of the [K^+^]. All the results above indicate that the PEG molecules do not adsorb on the surface of GB, but are left in the interstitial liquid phase as free polymers. 

It should be mentioned here that the specific reason for the non-adsorption of PEG on the surface of GB is still unclear. It may be caused by the short storage time (7 min in this study but many hours in the other studies [24,25]), or the big particle size of GBS (several micrometers in our study but nanometers in the other studies [24,25]). Consequently, the probability of PEG adsorption is significantly decreased during the rheological measurement period. Compared to the non-adsorption of PEG during the initial period, the adsorption of PCE can be achieved because of the following two possible reasons. On the one hand, PCE can be adsorbed by the side chain, which has the same structure as that of PEG, but the adsorption probability of PCE is significantly increased, because there are many side chains in one PCE molecule. On the other hand, as shown in Figure 1, the surface of GB is full of calcium, and some calcium ions are present in the interstitial liquid phase [18], which can interact with the carboxylate groups contained in PCE and then lead to the adsorption of PCE through its main chain [29].

### 3.3. Mixtures of Negatively Charged PCE and the Neutral PEG 

**Figure 8:**
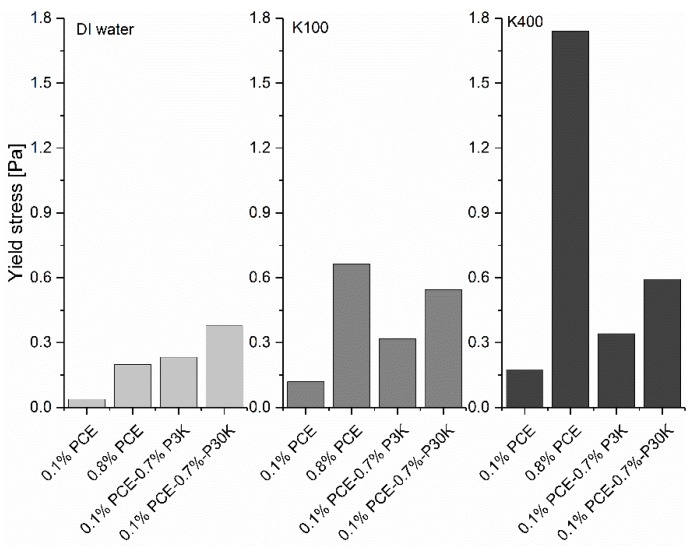
Yield stress of GBS with the addition of PCE and PCE/PEG mixtures with different [K^+^].

**Figure 9:**
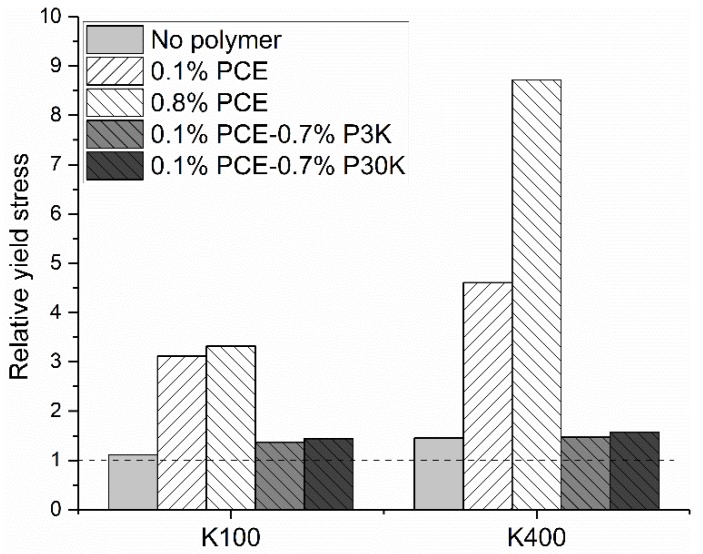
Relative yield stress of GBS, with the addition of different polymers with increasing [K^+^].

After showing the effect of pure polymer solutions (PCE and PEG) alone on the yield stress of GBS, in the next step, the effect of mixtures of PCE and PEG on the yield stress of GBS is considered. 0.7 % PCE (about 30 kDa) were replaced by PEG, either P3K or P30K. In this case, the total polymer dosage is still 0.8 %, and the effect of different charged polymers on the yield stress can be compared. The results are shown in Figure 8. The original shear rate and shear stress curve can be found in Figure S4. The relative yield stress with different [K^+^] was also calculated and is shown in Figure 9. 

Compared to the sample with PCE at a dosage of 0.1 %, increasing polymer dosage, regardless of PCE or PEG, results in higher yield stress with varied [K^+^], which indicates that the increasing free polymer amount in the interstitial liquid phase leads to the higher yield stress of GBS. Comparing the results obtained with different types of polymers at the same dosage of 0.8 %, clearly the effect of a polymer on the yield stress of GBS is related to the [K^+^] in the interstitial liquid phase. Without [K^+^], the GBS with the mixture of 0.1 % PCE and 0.7 % PEG shows higher yield stress (about 0.4 Pa) compared to that with 0.8 % PCE (about 0.2 Pa), while the opposite phenomena can be found with increasing [K^+^]. In addition, the PEG with higher molecular weight shows a stronger capability of increasing the yield stress than that with low molecular weight, which matches well with the previous research results [30]. If we compare the relative yield stress with the two [K^+^], as shown in Figure 9, it is found that a sample with 0.8 % PCE shows the highest relative yield stress, followed by the sample with PCE at the dosage of 0.1 %. The sample with the addition of the mixture of PCE and PEG shows the lowest relative yield stress. In the end, GBS with 0.8 % PCE shows the highest yield stress, compared to the other samples with [K^+^] of 400 mmol/L. 

### 3.4. Interparticle Forces with Negatively Charged PCE

**Figure 10:**
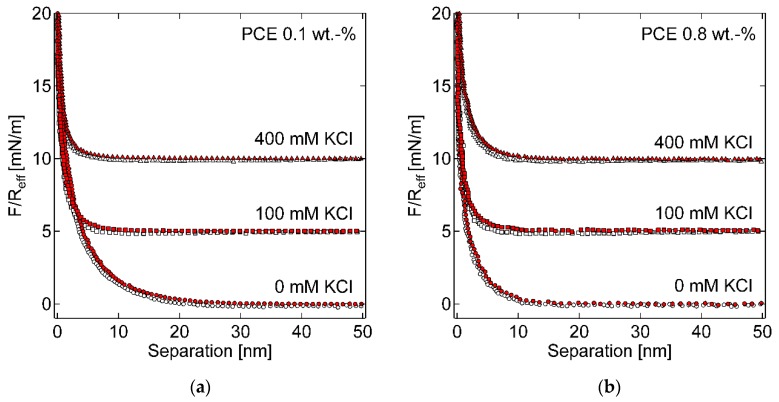
Measured force-distance curves for approach (filled symbols) and retraction (open symbols) in systems with (**a**) 0.1 %, (**b**) 0.8 % PCE and 0, 100, 400 mM KCl. Force distance curves are vertically shifted for clarity.

As the rheological performance of GBS is closely related to the interparticle force, in this section, the interparticle force between two glass beads, which are covered by the adsorbed PCE, are directly measured in different bulk solutions. As shown in Figure 10, clearly, through the adsorption of PCE, the interparticle forces between two glass beads in all solutions are changed into repulsive, and no adhesion can be found (without PCE it is adhesion, which is not shown here). In addition, for the system without KCl, a stronger repulsive force can be measured in the system with 0.1 % PCE than that with 0.8 % PCE. Furthermore, the increase of KCl concentration reduces the repulsive force, which results in a faster decay of the force.

**Figure 11:**
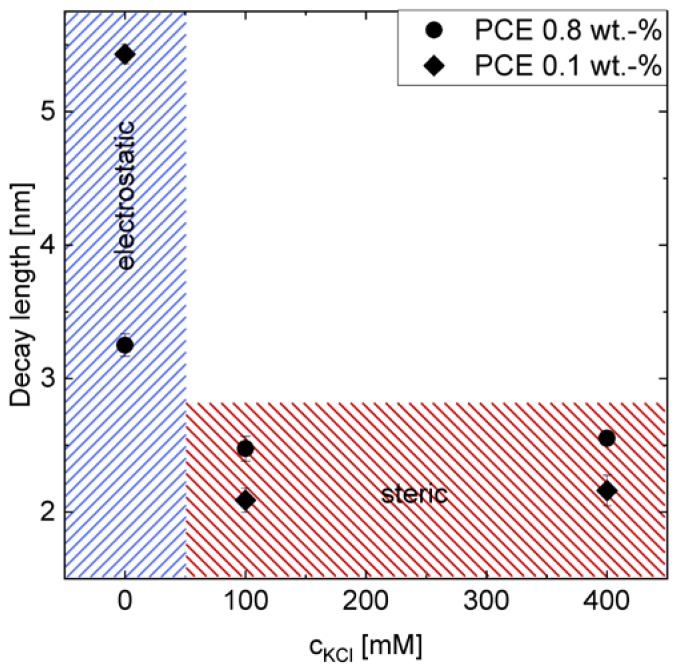
Decay length of approach curves for 0.1 % and 0.8 % PCE containing 0, 100, 400 mM KCl. No KCl implies electrostatic interaction, whereas for 100 mM and 400 mM, the Debye length is smaller than 1nm, implying steric interaction between the adsorbed PCEs side chains at the surfaces during approaching.

The decay of the force can be described by a mono-exponential function for separations larger than 2 nm, as shown in Figure 11. For the system with 0 mM KCl, the decay length in bulk solution with 0.1 % PCE is longer than that with 0.8 % PCE. The addition of KCl reduces the decay length for both PCE concentrations. However, the amount of KCl, i.e., 100 mM or 400 mM, has no influence on the decay length. Therefore, it is assumed that the reduction in decay length from 0 mM to 100 mM or 400 mM is induced by the additional ions in the system. The decay length at 0 mM can be thought of as the Debye length. 

For the system with KCl of 100 mM and 400 mM, the theoretical Debye lengths in both cases are smaller than 1 nm. However, the measured decay lengths are larger than 2 nm. Therefore, it is assumed that, for this concentration, the decay length is not described by the electrostatic interaction, but mainly by steric interaction of the adsorbed PCE. This also fits the fact that the decay length is independent of the ion concentrations, i.e., 100 mM or 400 mM in this study. It should be mentioned here that the systems containing 0.8 % PCE show higher noise in the baseline compared to that with 0.1 % PCE, which may result from the disturbance of the laser by the increased turbidity of these systems under high PCE concentration. Due to the turbidity of the solution, depletion forces were not accessible by AFM in this study, since the noise of the baseline was higher than the typical amplitudes of the depletion force [31].

## 4. Discussion

As shown in the results above, the addition of PCE firstly decreases the yield stress of GBS and then increases it along with the increasing dosages. Furthermore, even though the increasing adsorbed amount of PCE on the surface of GB can be found along with the increasing PCE dosage, most of the PCE is left as non-adsorbed polymers in the interstitial liquid phase at the dosage of 0.8 %, which can reach a concentration of 28.1 g/L. As the adsorption of PCE can trigger high steric hinderance force, it is reasonable to assume that the decrease of the yield stress through the addition of PCE is originated from the adsorption of PCE on the surface of GB. Along with the further increasing dosage of PCE, it is interesting to note that the yield stress gradually increases, which indicates that the adsorption of PCE does not play a dominant role at this stage. H. Bessaies-Bey [11] reports an attractive depletion force caused by the free polymers, and their corresponding effect on the increasing yield stress of cement paste. Normally, depletion forces occur when a polymer depletion zone exists around the particles, in which the polymer concentration is lower than in the bulk solution. When the separation of the particles is less than the diameter of the free polymer coil, a region of pure solvent is formed in the interstices between particles, which leads to an attraction between the particles, due to the difference in osmotic pressure between the polymer-depleted region and the bulk polymer solution. Hence, in this study, the further increase in the yield stress of GBS is likely to be related to the increasing attractive depletion force caused by the increasing non-adsorbed PCE concentration in the interstitial liquid phase, when the PCE dosage is higher than 0.1 %. Following this direction, as PEG does not adsorb, the added PEG molecules can only become free polymers in the interstitial liquid phase to raise the depletion force and then a monotonous increase in yield stress of GBS can be observed with the increasing PEG dosage. In addition, because the depletion force increases with the molecular weight of polymer [30], the GBS with P30K shows higher yield stress than P3K.

Regarding the effect of [K^+^], the negatively charged PCE can significantly increase the yield stress of GBS, along with the increasing [K^+^], but the neutral PEG does not have this effect, which indicates that non-adsorbed PCE molecules with a negative charge can interact with potassium ions but the neutral PEG molecules cannot. Afterwards, the question is raised how the different types of free polymers, namely the negatively charged PCE and neutral PEG, affect the yield stress of GBS with different [K^+^]. The comparison of the PCE and the mixture of PCE and PEG under the total polymer dosage of 0.8 % is discussed below.

For the mixture of negatively charged PCE and the neutral PEG, the added 0.7 % PEG cannot be adsorbed on the surface of GB, but becomes free polymers in the interstitial liquid phase. As the continuous adsorption of PCE from 0.1 % to 0.8 %, the sample with 0.8 % PCE should have a lower free polymer concentration (28.1 g/L) in the interstitial liquid phase, compared to that with the mixture of 0.1 % PCE and 0.7 % PEG (32.5 g/L). According to the model proposed by Asakura and Oosawa [30], in the dilute system, the depletion force depends on the number density of polymer molecules in the bulk solution, temperature, polymer radius of gyration (Rg), particle diameter and the distance between adjacent particles. As the same materials and experimental conditions were used during measurement and a similar Rg of PCE and P30K (7.29 nm and 8.54 nm for PCE and PEG, respectively, under pH = 11), the depletion force should be only related to the polymer number density. Consequently, as the similar molecular weight of PCE and P30K, GBS with 0.8 % PCE should always have lower yield stress than that with the mixture of 0.1 % PCE and 0.7 % PEG, if the effect of free polymer on the yield stress is dominated by the triggered depletion force. However, this is not the case in our study because the sample with PCE at a dosage of 0.8 % shows the highest yield stress with [K^+^] of 100 mmol/L and 400 mmol/L, but not for the reference sample. It indicates that the increased yield stress of the sample with the addition of PCE not only depends on the depletion force caused by the non-adsorbed PCE molecules in the interstitial liquid phase, but also the interaction of these negatively charged non-adsorbed PCE molecules, with cations in the solution. In addition, it is also reported that the depletion force caused by the charged macromolecules is much larger than that from non-charged macromolecules with the same polymer concentration [30], but the further addition of salts can rapidly decrease this force. If so, it is then expected that a sample with 0.8 % PCE should have higher yield stress than the mixture with 0.1 % PCE and 0.7 % PEG. The increasing [K^+^] in solution should gradually decrease the yield stress of the sample with 0.8 % PCE. However, as clearly shown in Figure 8, this is also not the case in our study, which again stresses the important effect of the interaction of charged polymers with the counterions on the rheological performance of GBS.

Afterwards, the comparison of results from AFM measurement and the rheological experiments is made, with an expectation of a better understanding on the macroscopic yield stress of GBS from the interparticle interaction on the microscale.

Normally, a stronger repulsive force in the AFM results means a higher stabilizing ability of suspension. In addition, a higher decay length indicates the longer effective domain for the repulsive force. In our AFM measurement, as stated above, the increase of KCl concentration in bulk solution reduces the repulsive force and results in a faster decay of the force. It corresponds well with the rheological experiments shown in Figure 3, in which an obvious increase of the yield stress of GBS can be observed with increasing [K^+^], regardless of the PCE dosage.

If we compare the effect of PCE, on the one hand, only repulsion, no adhesion, can be measured in the AFM measurement, which indicates that adhesion does not play a role here and the increasing yield stress of GBS, along with increasing [K^+^] under certain PCE dosage, is only caused by the decreasing repulsion. On the other hand, a lower repulsion is observed in AFM measurements by increasing the amount of PCE if there is no KCl present, which may result from the shorter Debye length, due to the screen of the increasing PCE concentration. However, this effect is of minor importance in the rheological measurement, since the pore solution already contains one order of magnitude higher ion concentration than by the addition of PCE. In the AFM measurement, a higher decay length can be found for samples with 0.8 % PCE than 0.1 % PCE under the KCL concentration of 100 mM and 400 mM, which is assumed to be caused by the higher steric force, due to the higher PCE adsorption amount under 0.8 % PCE. However, in the rheological experiment, higher yield stress of GBS can always be found with 0.8 % PCE, which seems to be contrary to the AFM results. It indicates again that the depletion force should play a more important role under high PCE dosage, but unfortunately, it cannot be measured by the AFM measurement, due to the turbidity of the solution. 

## 5. Conclusions

In this study, the effect of different charged polymers on the yield stress of glass bead suspension (GBS), with and without the presence of potassium ions, are evaluated. Furthermore, the effect of different amounts of polymer and [K^+^] in the bulk solution on the interaction forces between glass beads with adsorbed PCE is investigated by CP-AFM. The following conclusions can be obtained.

(1) In contrast to the first decrease and then increase in the yield stress of GBS with the increasing addition amount of PCE [18], a monotonous increase of yield stress can be observed with the increasing dosage of PEG, which is assumed to be caused by the depletion force, due to the non-adsorbing property of PEG on the surface of GB.
(2) The effect of free polymer in the interstitial liquid phase on the yield stress of GBS is related to its charge properties and, at the same time, the [K^+^] in solution. Along with the increasing [K^+^], the negatively charged PCE can significantly increase the yield stress of GBS, but the neutral PEG does not have this effect, which indicates that a non-adsorbed PCE molecule with a negative charge can interact with potassium ions and then significantly improve the attractive force, but PEG cannot. GBS with 0.8 % PCE shows the highest yield stress with [K^+^] of 400 mmol/L, even though fewer polymers are left in the interstitial liquid phase compared to samples with the addition of 0.1 % PCE and 0.7 %PEG. It indicates that, besides the attractive depletion force caused by the free polymer in the interstitial liquid phase, the interaction of charged polymers with the counterions also plays important role in affecting the rheological performance of GBS.
(3) A close relationship between the interparticle force on the microscale and the macroscopic rheological performance of GBS can be found. Specifically, the increasing KCl concentration reduces the repulsive force between GB with adsorbed PCE. Regarding the effect of PCE, a lower repulsion is observed by increasing the PCE amount with KCl of 0 mM. A higher decay length can be found for samples with 0.8 % PCE than 0.1 % PCE, under the KCL concentration of 100 mM and 400 mM. It indicates again that the depletion force should play an important role under a high PCE dosage, but unfortunately, it cannot be measured by the AFM measurement in this study, due to the turbidity of the solution.

## Supplementary Materials

The following are available online at https://www.mdpi.com/1996-1944/13/7/1490/s1, Figure S1: pH of GBS with the addition of different polymers under increasing [K^+^], Figure S2: Profile of the rheological measurement, Figure S3: Measured shear stress *vs* shear rate curve and the corresponding fitting curve, Figure S4: Shear stress *vs* shear rate curve of the samples with the addition of polymers under increasing [K^+^].

## Abbreviation

Abbreviation	Full name
PCE	Polycarboxylate superplasticizer
PEG	Polyethylene glycol
SEM	Scanning electron microscope
P3K	PEG with the molecular weight of 3,000 Da
P30K	PEG with the molecular weight of 30,000 Da
GBS	Glass bead suspension
GB	Glass bead
SP	Superplasticizer
SCC	Self-compacting concrete
UHPC	Ultra-high performance concrete
w/c	Water to cement ratio
CP-AFM	Colloidal probe atomic force microscope
DI water	Deionized water
KCl	Potassium chloride
TOC	Total organic carbon
bwog	By weight of glass beads
Rg	Polymer radius of gyration
