# Supplementary material for: Interaction of Different Charged Polymers with Potassium Ions and Their Effect on the Yield Stress of Highly Concentrated Glass Bead Suspensions
Source: Materials (Basel). 2020 Mar 25;13(7):1490. doi: 10.3390/ma13071490 (PMC7177864; doi:10.3390/ma13071490)
Supplement: Supplementary file 1 [file materials-13-01490-s001.pdf]

materials

MDPI

Supplementary Materials

Interaction of Different Charged Polymers with Potassium Ions and Their Effect on the Yield Stress of Highly Concentrated Glass Bead Suspensions

Zichen Lu [1,*], Simon Becker [2], Sarah Leinitz [3], Wolfram Schmidt [3], Regine von Klitzing [2], Dietmar Stephan [1,*]

[1] Department of Civil Engineering, Technische Universität Berlin, Berlin 13355, Germany
[2] Department of Physics, Technische Universität Darmstadt, 64289, Germany; becker@fkp.tu-darmstadt.de (S.B.); klitzing@smi.tu-darmstadt.de(R.v.K.)
[3] Bundesanstalt für Materialforschung und - prüfung (BAM), Berlin 12205, Germany; sarah.leinitz@bam.de(S.L.); Wolfram.Schmidt@bam.de(W.S.)
* Correspondence: zichen.lu@tu-berlin.de(Z.L.); stephan@tu-berlin.de(D.S.)

Received: 16 February 2020; Accepted: 23 March 2020; Published: 23 March 2020

Figure S1 shows the pH of glass bead suspension (GBS) with the addition of different polymers under increasing [K^+]. Normally the pH of the prepared GBS was in the range of 10.1 to 11.3. However, it also depends on the concentration of salts and polymers.

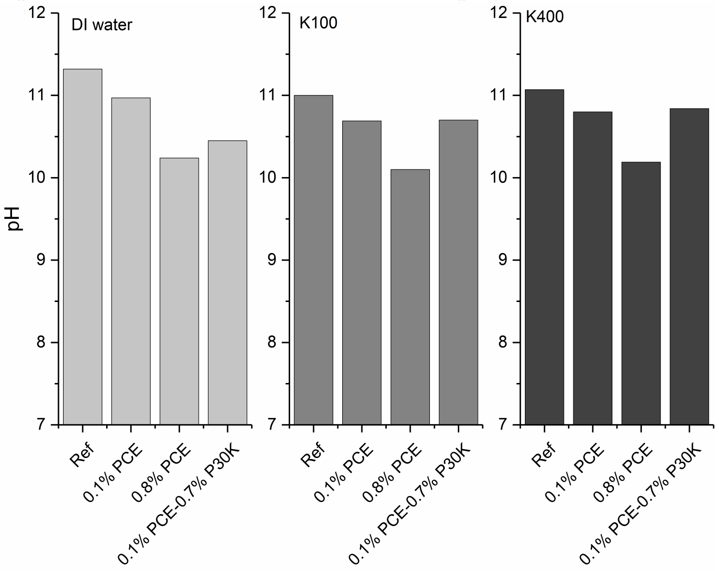

Figure S1. pH of GBS with the addition of different polymers under increasing [K^+].

As shown in Figure S2, the GBS was measured rotational with a stepwise increased shear rate from 0 s^{-1} to 8.378 s^{-1}. The increase of the shear rate took place every 15 seconds. After reaching 8.378 s^{-1}, the shear rate was decreasing also stepwise in 14 steps. The total measurement time is 7 min.

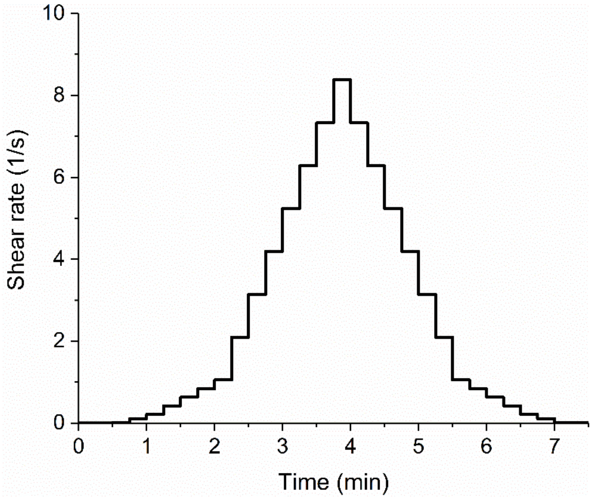

Figure S2. Profile of the rheological measurement.

After each measurement, a curve showing the change of shear stress along with the shear rate could be obtained. Herschel Bulkley's equation ($\tau = \tau_0 + k\dot{\gamma}^n$) was used to fit the obtained curve and then the information on yield stress and viscosity of GBS could be gotten. One example of fitting and the corresponding R-square are shown in Figure S3. We can find that a good fitting was obtained.

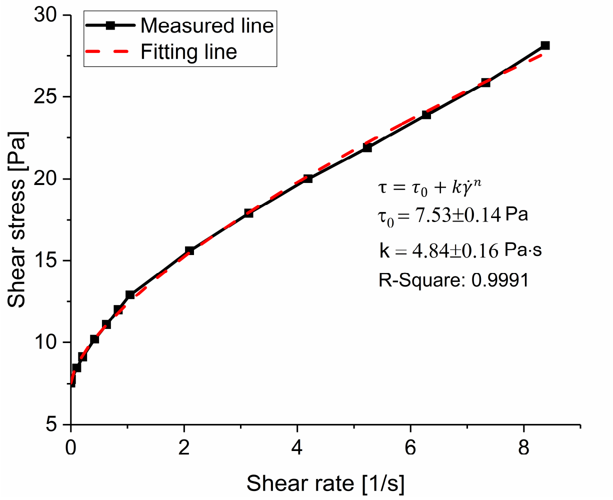

Figure S3. Measured shear stress vs shear rate curve and the corresponding fitting curve.

The shear stress and shear rate curve of GBS with different types of polymers under increasing [K^+] can be found in Figure S4. Each curve was fitted as the method shown in Figure S3 and then the yield stress was obtained. The results are shown in Fig. 8 in the manuscript.

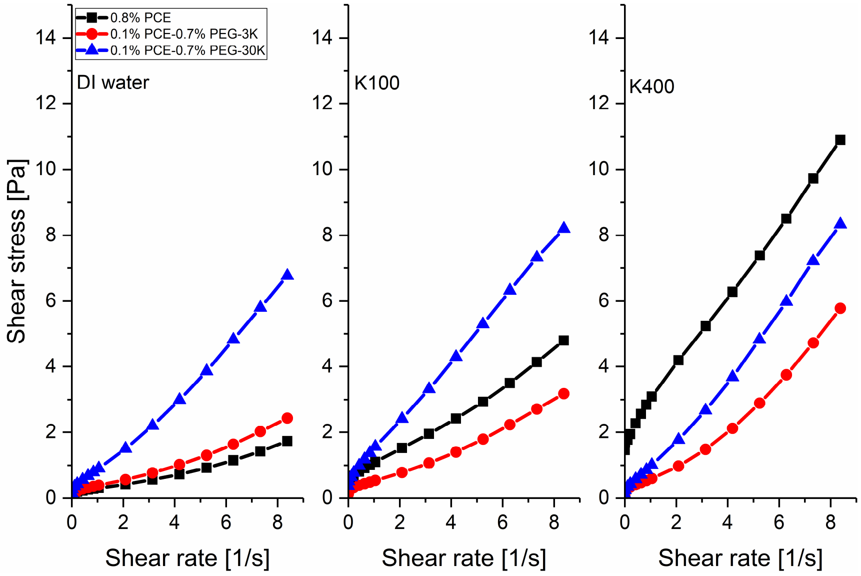

Figure S4. Shear stress vs shear rate curve of the samples with the addition of polymers under increasing [K^+].

© 2020 by the authors. Submitted for possible open access publication under the terms and conditions of the Creative Commons Attribution (CC BY) license (http://creativecommons.org/licenses/by/4.0/).
